# Home range, habitat selection, density, and diet of golden jackals in the Eastern Plains Landscape, Cambodia

**DOI:** 10.1093/jmammal/gyab014

**Published:** 2021-03-20

**Authors:** Jan F Kamler, Christin Minge, Susana Rostro-García, Tazarve Gharajehdaghipour, Rachel Crouthers, Visattha In, Chen Pay, Chanratana Pin, Prum Sovanna, David W Macdonald

**Affiliations:** 1 Wildlife Conservation Research Unit, University of Oxford, Department of Zoology, The Recanati-Kaplan Centre, Tubney House, Abingdon Road, Tubney, Abingdon OX13 5QL, United Kingdom; 2 Institute of Ecology and Evolution, Friedrich-Schiller University of Jena, 07443 Jena, Germany; 3 Department of Natural Resources Management, University of British Columbia, 2424 Main Mall, Vancouver, British Columbia V6T 1Z4, Canada; 4 World Wild Fund for Nature Cambodia, Street 322, Phnom Penh 12302, Cambodia; 5 Ministry of Environment, 48 Samdach Preah Sihanouk Blvd., Phnom Penh 12301, Cambodia

**Keywords:** biomass consumed, *Canis aureus*, food habits, prey selection, processional termites, Srepok Wildlife Sanctuary

## Abstract

We used radiocollars and GPS collars to determine the movements and habitat selection of golden jackals (*Canis aureus*) in a seasonally dry deciduous forest with no human settlements in eastern Cambodia. We also collected and analyzed 147 scats from jackals to determine their seasonal diet and prey selection. The mean (± *SE*) annual size of home-range ranges (47.1 ± 2.5 km^2^; *n* = 4), which were mutually exclusive between mated pairs, was considerably larger than that previously reported for this species, resulting in an extremely low density (0.01 jackal/km^2^). The unusually large home ranges and low density probably were due to the harsh dry season when most understory vegetation is burned and nearly all waterholes dry up, thereby causing a large seasonal decline in the availability of small vertebrate prey. Resident groups consisted of an alpha pair, but no betas, and were situated only in areas not occupied by leopards (*Panthera pardus*) and dholes (*Cuon alpinus*). Jackals avoided dense forests and streams, and had a strong selection for dirt roads, possibly to avoid larger predators. Overall the jackal diet was diverse, with at least 16 prey items identified, and there was no significant difference in diet composition between the cool-dry and hot-dry seasons. Scat analysis showed that the main food items consumed by jackals were processional termites (*Hospitalitermes* spp.; 26% biomass consumed), followed by wild pig (*Sus scrofa*; 20%), muntjac (*Muntiacus vaginalis*; 20%), and civets (17%). Compared to available biomass, jackals were not random in their consumption of ungulates because muntjac were selectively consumed over larger ungulate species. Dietary overlap with dholes and leopards was relatively low, and consumption patterns indicated jackals were preying on ungulates rather than scavenging from kills of larger carnivores. Our results showed that the jackal is an extremely adaptable and opportunistic species that exhibits unique behaviors to survive in an extreme environment near the edge of its distribution.

The golden jackal (*Canis aureus*; hereafter jackal) is a mesocanid that is widely distributed across Eurasia, from an expanding population in Europe, across southern Asia to Indochina (Hoffmann et al. 2018). The jackal once was thought to occupy eastern and northern Africa, although this mesocanid is now classified as the African wolf (*C. lupaster* or *C. anthus*—Rueness et al. 2011; Koepfli et al. 2015; Viranta et al. 2017). The status of jackals in Vietnam is uncertain because the species has not been recorded there since 2004 (Hoffmann et al. 2019); eastern Cambodia therefore is at or near the known eastern limit of their distribution. The jackal is an opportunistic and generalist species that can live in a wide variety of habitats, and consume a wide variety of foods (Hayward et al. 2017; Hoffmann et al. 2018). In fact, moderate levels of human activity and habitat transformation appear to benefit jackals, primarily because of increases in food resources such as carrion, refuse, and crops (Hoffmann et al. 2018). In addition, human persecution of apex carnivores, especially gray wolves (*Canis lupus*), seem to benefit jackals, and the extermination of wolves has been identified as one of the reasons for the expansion of jackals across Europe (Krofel et al. 2017; Newsome et al. 2017).

Most studies of jackal ecology are biased toward environments and food resources that are heavily impacted by humans. For example, one of the most important food sources of jackals in Europe are slaughter remains and other animal waste from livestock (Supplementary Data SD1), which represents about 40% of the jackal diet across the continent (Ćirović et al. 2016). Similarly, in some localities in Europe, wild ungulate carrion left by human hunters is the most important food source of jackals (Lanszki et al. 2018; Supplementary Data SD1). The same is true in countries across Asia, where livestock carrion, poultry carrion, and refuse, are important parts of jackal diets (Macdonald 1979; Rotem et al. 2011; Supplementary Data SD1). Even within protected areas of India, livestock carrion often is an important component of jackal diets (Supplementary Data SD1). Consequently, there has been little research on the diet and ecological niche of jackals in natural areas with no human-generated foods. In particular, the role of jackals as predators of ungulates, or scavengers of carcasses, in natural areas is not well known or understood.

In a review of jackal diets, Hayward et al. (2017) found that jackals significantly preferred brown hares (*Lepus europaeus*; 4 kg), and that their preferred prey weight range was 0–4 kg. This conclusion is supported by several diet studies that showed the main prey of jackals is small mammals (Supplementary Data SD1). In contrast, Hayward et al. (2017) found black-backed jackals (*Canis mesomelas*), a similar species, significantly preferred small (< 30 kg) ungulate species that hide their young, and that their preferred and accessible weight prey range was 14 – 26 kg. This conclusion was in agreement with previous studies showing black-backed jackals were major predators of small- and medium-sized ungulates in relatively natural areas (Wyman 1967; Klare et al. 2010). Similarly, other mesocanids, such as African wolves and coyotes (*C. latrans*), were shown to be major predators of small- and medium-sized ungulates in relatively natural areas (Bekoff and Gese 2003; Moehlman and Hayssen 2018). Reasons for these apparent differences in niche and predatory behavior between golden and black-backed jackals found by Hayward et al. (2017) are not known, but results possibly could have been biased because of a lack of diet studies of golden jackals from natural areas where human-generated food was absent.

In Asia, several dietary studies showed that jackals consume wild ungulates, presumably as carrion (Supplementary Data SD1). However, some studies in Indian and Sri Lankan protected areas indicated that jackals were preying on wild ungulate fawns, including those of chital (*Axis axis*), blackbuck (*Antilope cervicapra*), and nilgai (*Boselaphus tragocamelus*—Eisenberg and Lockhart 1972; Supplementary Data SD1). This indicates that golden jackals might prey more on wild ungulates than generally is believed, and that they might have a similar predatory niche as that of black-backed jackals, African wolves, and coyotes, at least in natural areas with little or no human-generated foods.

Although jackals readily scavenge livestock carrion and human-killed wild ungulates, the frequency with which jackals scavenge ungulates killed by larger carnivores is not well documented. Presumably, jackals are unlikely to be major scavengers of wolf-killed ungulates in Europe, given that gray wolves kill and spatially displace jackals (Giannatos 2004; Krofel et al. 2017; Mohammadi et al. 2017; Newsome et al. 2017). Similarly, tigers (*Panthera tigris*) sometimes eat jackals (Heptner and Sludskii 1992; Simcharoen et al. 2018), and Schaller (1967) stated that jackals in India avoided tiger-killed ungulate carcasses, even if they were common. In addition, jackals are unlikely to scavenge frequently from ungulates killed by leopard (*P. pardus*), given the propensity of leopards to kill and consume jackals (Lukarevsky 1988; Meena et al. 2013; Simcharoen et al. 2018; Kamler et al. 2020b), including when jackals visit carcasses (Schaller 1967). Jackals therefore might spatially avoid areas that frequently are used by larger carnivores, similar to what has been reported between other carnivores (Kamler et al. 2013). Nevertheless, other investigators have stated that jackals scavenge from kills of tigers, dholes (*Cuon alpinus*), and wolves in India (Jhala and Moehlman 2004), and other mesocanids seem to scavenge readily kills of larger carnivores (Lamprecht 1978; Bekoff and Gese 2003; Moehlman and Hayssen 2018). To gain a better understanding of the ecological role of jackals as scavengers in natural areas lacking human-generated food, more information is needed about their diet from areas where they are sympatric with apex carnivores.

Studies documenting the space use of jackals in Eurasia are much fewer than diet studies, but similarly are biased toward areas heavily impacted by humans. Jackals were found to have relatively small home ranges of 0.1 – 14.3 km^2^ on farmland near villages or in areas where jackals were provisioned with carrion (Macdonald 1979; Poché et al. 1987; Giannatos 2004; Aiyadurai and Jhala 2006; Jeager et al. 2007; Rotem et al. 2011). The few studies from relatively undisturbed natural areas indicate jackal home ranges are considerably larger (21.2 – 34.8 km^2^—Rotem et al. 2011; Charaspect et al. 2019) than in human-dominated sites, probably because of reduced availability of human-generated foods. Similarly, coyotes were found to have smaller home ranges in urban compared to rural areas across their distribution, owing to the higher food resources and lower mortality in urban areas (Gehrt and Riley 2010). In contrast, other studies on coyotes, black-backed jackals, and African wolves have shown them to live in relatively small (< 10 km^2^) home ranges within natural areas, probably due to abundant food resources in at least some natural areas (Kamler and Gipson 2000; Moehlman and Hayssen 2018; Kamler et al. 2020c). Additional studies of jackal space use are needed from relatively undisturbed natural areas to better understand the movements and land requirements of this species in environments not heavily impacted by humans and where human-generated food is absent.

We studied the ecology of jackals in the core zone of Srepok Wildlife Sanctuary (SWS), within the Eastern Plains Landscape, Cambodia, which is located near the eastern limit of the jackal distribution. There are no villages or agricultural fields within the core zone of SWS, and livestock grazing is prohibited; this jackal population therefore was not influenced by human settlements or human-generated foods. Our main objectives were to determine the home ranges, habitat selection, density, diet, and prey selection of jackals. We predicted that home ranges would be relatively large, and the density would be relatively low, given that this study occurred in a natural area. Because jackals are a habitat generalist (Hoffmann et al. 2018), we predicted they would use habitats in proportion to availability. On account of the lack of human-generated foods, we predicted that jackals would consume mostly small rodents and hares because these species are within the jackal’s estimated preferred prey weight range of 0–4 kg (Hayward et al. 2017), and that jackals would scavenge from kills of large carnivores.

## Materials and Methods

### Study area

**Fig. 1.**
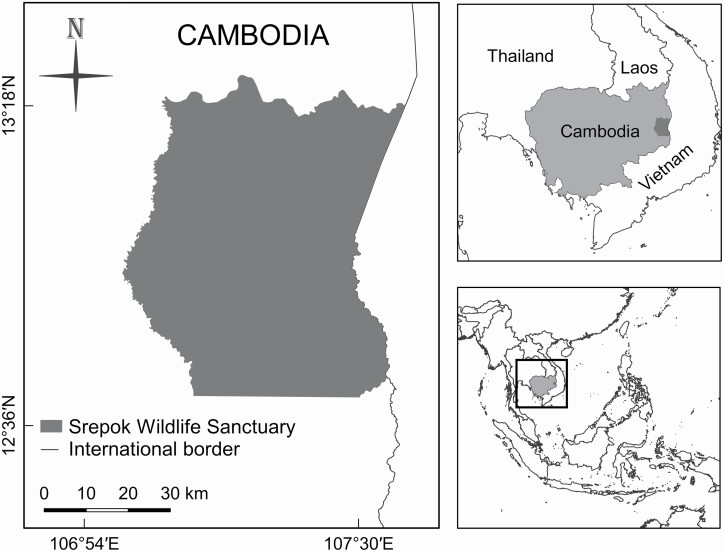
Location of Srepok Wildlife Sanctuary within Cambodia and the region.

We undertook the research in SWS (3,730 km^2^), formerly called Mondulkiri Protection Forest (until 2016), located in eastern Cambodia (Fig. 1). The habitat of SWS is dominated (ca. 70%) by open and seasonally dry deciduous forests (DDF) in relatively flat terrain, interspersed with small patches of mixed deciduous-evergreen forests on hilltops, and riparian forests along streams and rivers (Rostro-García et al. 2018). The DDF, also called dry dipterocarp forest, is dominated by two species of Dipterocarpaceae trees, *Shorea obtusa* and *Dipterocarpus tuberculatus*, and an understory of grasses and herbaceous bamboo (*Vietnamosasa* spp.—Pin et al. 2013). The SWS has a distinct dry season from about November to April (average monthly rainfall is 3 – 121 mm), with a pronounced rainy season from May to October (248 – 370 mm per month; rainfall data were from nearby Sen Monorom, Cambodia, 1982–2012; climate-data.org; accessed 10 July 2019). Frequent annual dry-season fires, both natural and human-caused, occur in the DDF after the dipterocarp trees lose their leaves, burning most of the grassy understory (McShea and Davies 2011). The DDF is well adapted for dry-season fires, and anthropogenic fires in the region can be traced back to the late Pleistocene (Wanthongchai and Goldammer 2011); thus, we considered the habitat in our study site natural even if some dry-season fires may have been caused by humans. The elevation ranges from 100 to 400 m. Large (> 15 kg) carnivores present in SWS during the study included the leopard, dhole, and sun bear (*Helarctos malayanus*—Rostro-García et al. 2018). Other smaller carnivores present during the study included the jungle cat (*Felis chaus*), leopard cat (*Prionailurus bengalensis*), yellow-throated marten (*Martes flavigula*), small Asian mongoose (*Herpestes javanicus*), crab-eating mongoose (*H. urva*), ferret-badger (*Melogale* spp.), large Indian civet (*Viverra zibetha*), small Indian civet (*Viverricula indica*), large-spotted civet (*Viverra megaspila*), and Asian palm civet (*Paradoxurus hermaphroditus*—Gray et al. 2014). The wild ungulate community in SWS is dominated by banteng (*Bos javanicus*), wild pig (*Sus scrofa*), and northern red muntjac (*Muntiacus vaginalis*), with very small numbers of Eld’s deer (*Rucervus eldii*), gaur (*Bos frontalis*), and sambar (*Rusa unicolor*—Gray et al. 2013). Our research was carried out in a core zone (ca. 1,700 km^2^), located in the eastern part of SWS, where human access is restricted. Within the core zone there were no villages or agricultural fields and livestock grazing was not permitted. Human activities within the core zone included law enforcement patrols, research activities, and collection of non-timber forest products by local people primarily during the dry season. The only permanent infrastructure within the core zone was three ranger stations that were permanently occupied by 2–8 rangers, and two ranger substations that were occasionally occupied. Refuse from the ranger stations were burned at each site; therefore, jackals on our study site did not have access to human-generated foods.

### Capture and monitoring

We captured jackals using padded foothold traps (Woodstream Corp., Lititz, Pennsylvania) set along dirt roads where we encountered numerous jackal sign (e.g., tracks and scats). Padded foothold traps were set with a high pan tension to exclude species smaller than jackals (Kamler et al. 2008). Trapping was undertaken only during the dry season to avoid capturing juveniles. We set traps just prior to sunset, then monitored the traps throughout the night using radiotelemetry trap monitors (Telonics, Inc., Mesa, Arizona), which emit a fast-paced signal when a trap is sprung. The signals from the trap monitors were checked every 30 min, then traps were closed at sunrise. Captured jackals were collared, sex determined, weighed to the nearest 0.1 kg, aged according to tooth wear and reproductive condition, then released immediately at the capture site. All captured jackals showed heavy wear on incisors (Gier 1968) and had large testes (*n* = 3 males) or dark elongated teats (indicating previous nursing; *n* = 3 females); thus, they were considered adults and classified as alphas following Kamler et al. (2019). During 2013, we fitted three captured jackals (one male, two females) with radiocollars (Advanced Telemetry Systems, Inc., Isanti, Minnesota) weighing 190 g. In 2014 and 2015, we fitted three captured jackals (two males, one female) with Iridium GPS collars (Tellus Light model; FollowIt, Lindesberg, Sweden) weighing 240 g. One female jackal was recaptured after the GPS collar expired, and she was refitted with a radiocollar so that we could continue to monitor her movements. We followed the animal care and use guidelines of the American Society of Mammalogists (Sikes et al. 2016) and our research protocol was approved by the Forestry Administration, Ministry of Agriculture Forestry and Fisheries, Phnom Penh, Cambodia.

One GPS collar placed on a female jackal (4-F) in 2014 was programmed to obtain one location every 24 h, whereas two GPS collars placed on male jackals in 2015 (5-M, 6-M) were programmed to obtain 4 locations/day, on average one location every 6 h. Radiocollared jackals were radiotracked 1–3 times per week during the night throughout the dry season by obtaining directional bearings using handheld “H” antennas and 3-element yagi antennas from motorcycles. When locating jackals, observers took ≥ 2 bearings from known telemetry stations < 5 min apart. We calculated location estimates using the maximum likelihood estimation option in the program Locate II (Pacer, Inc., Truro, Nova Scotia, Canada). Mean (± *SE*) error of estimated locations was 68.4 (± 7.5) m when using reference collars (*n* = 12) placed at known locations 1 – 2 km from observers (i.e., typical distance when tracking animals). Telemetry and GPS locations for jackals were assumed to be independent because only one location per animal was obtained during any 6-h period. Our study site became inaccessible during the rainy season because of high water levels and impassable rivers, so we could not obtain locations on radiocollared jackals during most of this season (June–October). However, locations from GPS-collared jackals were obtained during both the dry and rainy seasons.

### Home ranges and density

We estimated the home-range size of collared jackals using 95% fixed kernel density estimates (KDEs—Worton 1989). We also calculated 50% KDEs for jackals to represent their core areas, which are areas of concentrated use within home ranges (Kamler et al. 2003, 2012). To allow for comparisons with previous studies, we calculated home ranges and core areas using 95% and 50% minimum convex polygon (MCP), respectively. To determine if there were seasonal changes in home-range size, we calculated monthly home ranges using 95% KDE for the three jackals fitted with GPS collars. We did not calculate monthly home ranges for the radiocollared jackals because of low numbers of locations per month. We only calculated monthly home ranges of GPS-collared jackals if locations were obtained for at least 2 weeks within that month and had at least 28 locations. One jackal (4-F) went on an extraterritorial foray of 12 km during a single night; this movement was removed from home-range analysis (Kamler and Gipson 2000; Kamler et al. 2019). For the mated pair 4-F and 5-M, we measured overlap of intensity of use within home ranges using the utilization distribution overlap index (UDOI—Fieberg and Kochanny 2005) based on 95% and 50% UD isopleth levels (see Kamler et al. 2019 for more details). To allow comparisons with previous studies, we also calculated percent area overlap using 95% and 50% MCPs (Cole 1949; Kamler et al. 2019). Animal home ranges based on KDE and MCP were quantified using the rhr (Signer and Balkenhol 2015) and adehabitatHR (Calenge 2015) packages in R (R Core Team 2020), respectively. To outline a single contiguous 95% isopleth polygon indicative of a complete home range while preventing over-smoothing, we calculated Gaussian kernels using 0.8 × reference bandwidth (*h*_ref_—Kie et al. 2010), unless the *ad hoc* bandwidth suggested by Kie (2013) was greater than 0.8*h*_ref_, in which case we used the greater bandwidth estimated by the ad hoc technique.

**Fig. 2.**
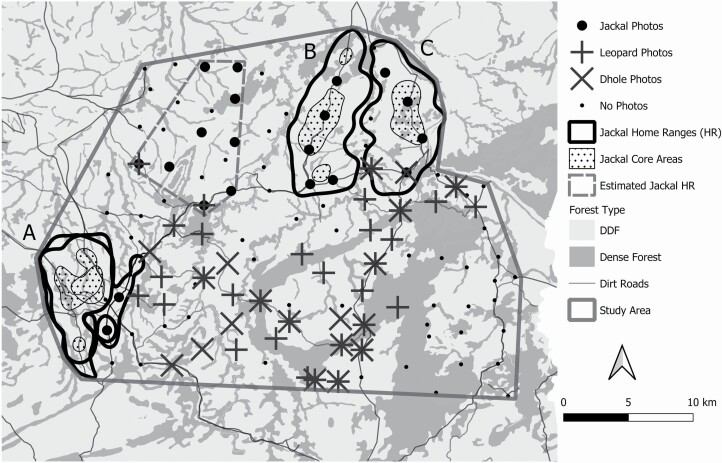
Home ranges (95% fixed kernel density estimates [KDEs]) and core areas (50% KDE) of four collared jackals in Srepok Wildlife Sanctuary, Cambodia. The home ranges represent: (A) the mated pair of 4-F and 5-M, (B) 6-M, and (C) 1-F. Also shown is the estimated home range of a fourth mated pair of jackals (dashed polygon), based on photographic records from camera traps. All photographic records of jackals, leopards, and dholes are shown to illustrate spatial partitioning between jackals and large carnivores. Our 800-km^2^ intensive study area encompassed where we trapped jackals, established scat transects, and carried out camera-trap surveys.

We estimated the pre-whelping (i.e., dry season) density of jackals in our study site based on the number of jackal groups present, multiplied by the mean number of adult animals per group, divided by the total area occupied by our study site. This method has been previously used to calculate the density of other *Canis* species, including gray wolves (Ballard et al. 1997), coyotes (Kamler and Gipson 2000), and black-backed jackals (Kamler et al. 2012). Mean number of adult jackals per group was determined by our observations, and those of other researchers, along with trapping results, and photographs from concurrent camera-trap surveys. Jackals were individually identified in the camera-trap photos based on collar type and color, or unique markings on their fur. Our intensive study area occupied an 800-km^2^ area within the core zone of SWS, where we trapped for jackals, established scat-transect lines, and carried out camera-trap surveys across several years (Fig. 2). The monitoring of collared jackals occurred from 2013 to 2016, whereas we monitored jackals during camera-trap surveys undertaken for leopards and their prey in 2014 (*n* = 42 stations), 2016 (*n* = 46 stations), and 2018 (*n* = 164 stations; see Rostro-García et al. 2018 for methodological details). Different-sized grids were used in different years, but in every survey, the camera stations were spaced 2–3 km apart along dirt roads and trails; therefore, we assumed that the surveys were adequate to detect any resident jackals that were present within the grids, particularly because jackals were attracted to dirt roads and used them extensively (see results of resource selection). We combined results from all 3 years of camera trapping to determine the area occupied by resident jackals. We considered a camera-trap location as occupied by jackals if it obtained > 1 independent photograph of jackals over the 2-month survey periods. Single photographs of jackals over a 2-month period likely represented dispersing jackals, or breeders taking extraterritorial forays, rather than areas permanently occupied by mated pairs. To compare spatial overlap of mated pairs of jackals and large carnivores, we compared locations where leopards and dholes were photographed across surveys to those occupied by jackals, including the jackal home ranges we calculated.

### Habitat selection

We used a resource selection function (RSF) to calculate the relative probability of use (selection versus avoidance) for collared jackals following a within home-range (third order) presence versus available design (Johnson 1980). We constructed RSFs using a generalized linear mixed model (GLMM) with a Bernoulli response (1 = presence, 0 = available) using Template Model Builder (TMB) via the glmmTMB::glmmTMB function in R (Brooks et al. 2017; R Core Team 2020). We constructed a 1:10 presence to available ratio (with a total number of 1,867 presence points) by randomly sampling the available habitat within each jackal’s home range (100% MCP) using ArcGIS. In addition to considering four continuous distance-based variables (distance to nearest dense [evergreen and semi-evergreen] forest patch, DDF, dirt road, and stream) as fixed effects, we also followed recommendations by Muff et al. (2020) and included random intercept (by ID) and slopes, with fixed intercept variance (10^6^) in our weighted logistic regression model (weight = 1,000). Prior to fitting our model, we log-transformed, centered and scaled (mean = 0, *SD* = 1) our variables, and tested for collinearity using Pearson’s *r*. We inferred selection when β coefficients were negative, and avoidance when they were positive.

We evaluated the predictive strength of our model using *k*-fold cross-validation (Boyce et al. 2002) blocked by random individual following the procedures outlined in Roberts et al. (2017). We used a blocked by individual design to prevent overconfidence in our model performance considering our small sample size. At each iteration, we withheld all locations from one individual, and used the remaining locations to train our model. We repeated this procedure iteratively until all data were cross-validated. We then used Spearman’s rho to measure correlation between the area-adjusted frequencies for each withheld set and 10 RSF equal-area bins (Boyce et al. 2002).

### Diet analysis and prey selection

The diet of jackals was determined by analysis of 147 scats (i.e., feces) that were collected during 2013 and 2014. Scats were collected along 30 transects (2 km each) that were established on dirt tracks and trails throughout the core zone of SWS, as well as opportunistically when carrying out other research. For each scat, the diameter, date, and GPS location were recorded. Scats of jackals were distinguished from other species based on size, placement, and DNA analysis of selected scats. Jackals typically defecate on elevated objects, such as small shrubs and tufts of grass, that line trails or dirt tracks (Macdonald 1979; Kamler et al. 2020a), rather than directly on the paths themselves. We took a random sample of 10 putative jackal scats collected from shrubs and tufts of grass, and sent them to the Sackler Institute for Comparative Genomics, American Museum of Natural History (New York), who confirmed that the scats were from jackals based on mitochondrial DNA analysis as described by Caragiulo et al. (2014). Concurrent diet studies used DNA analysis to confirm that other carnivore scats collected in SWS belonged to leopards (*n* = 73 scats—Rostro-García et al. 2018), dholes (*n* = 165—Kamler et al. 2020d), jungle cats (*n* = 16) and leopard cats (*n* = 130—Rostro-García et al. In press), none of which were found to defecate on small shrubs or tufts of grass. We therefore were confident that all the carnivore scats collected from small shrubs and tufts of grass along trails and dirt tracks were from jackals.

We washed each scat, then separated and identified remains of different prey items using a reference collection. For small rodents, it generally was not possible to identify remains to species given the great diversity of small rodents that potentially occur in the area (at least 27 species in 16 genera—Lunde and Son 2001) and the fragmentary remains found in the scats. For larger mammals, hair samples from each scat were identified to species where possible, by examining the structures of the cuticle and medulla under a microscope, and comparing those to the medullas of hairs from known species. We could not reliably distinguish between the hair structures of the four civet species; therefore, we grouped all civet hair into one category. We visually estimated the percentage of each prey item in a scat, but excluded prey items that were considered trace (i.e., 1 – 2% of scat) to minimize bias (Kamler et al. 2007). The dry weight was determined by weighing each scat to the nearest 0.1 g after washing in a sieve and drying.

We quantified results from scat analysis in terms of the percent biomass consumed because it is ecologically the most relevant parameter (Klare et al. 2011a). Following the recommendations of Klare et al. (2011a), we also calculated percent volume, and the frequency of occurrence (i.e., percentage of scats containing a particular food item) to make our results comparable to previous studies. To calculate the percent biomass of consumed items, we used correction factors (CFs) developed for red foxes (*Vulpes vulpes*—Goszczyński 1974; Jedrzejewska and Jedrzejewski 1998) and used previously on black-backed jackals (Klare et al. 2010; Kamler et al. 2012). Based on feeding trials, the CFs convert the weight of dry prey remains in scats to the estimated amount of biomass consumed for each prey species (Locke 1961; Goszczyński 1974). Following Goszczyński (1974) and Locke (1961), we used the following CFs: muntjac (118), wild pig (84), medium-sized (1 – 10 kg) mammal (50), bird (35), small (< 1 kg) mammal (23), herpetofauna (18), egg shell (15), seeds (14), termite (12), and beetle/crab (5). For ungulates, we used a CF of 118 for muntjac because this was value determined by Goszczyński (1974) for the consumption of roe deer (*Capreolus capreolus*; 15 – 35 kg—Macdonald and Barrett 2002), which is similar in size to muntjac (20 – 28 kg—Francis 2008); we hypothesize that canids should consume the carcasses of both ungulates in a similar manner. For wild pigs, we assumed jackals were consuming piglets and young individuals, because we found piglet hooves within jackal scats. We therefore used a CF of 84 for wild pigs, assuming jackals consumed individuals with a body mass of about 15 kg, which was between adult muntjac and medium-sized mammals. Based on the biomass of prey categories consumed, we calculated Levin’s measure of niche breadth (*B*—Krebs 1989). We used results from concurrent dietary studies in SWS of leopards (Rostro-García et al. 2018) and dholes (Kamler et al. 2020d) to calculate the degree of dietary overlap between jackals and both large carnivores using Horn’s index of overlap (*R*_0_—Krebs 1989) based on biomass consumed. Results of dietary overlap would show potential competition for prey species, and help determine if jackals were scavenging from ungulates killed by both large carnivores.

To determine prey selection of ungulate species consumed by jackals, we calculated Jacobs’ (1974) electivity index *D* to assess which prey species were selected (0 < *D* ≤ 1) and which were avoided (−1 ≤ *D* < 0) based on biomass consumed versus biomass available. To determine biomass available for each ungulate species, we multiplied adult female weights (i.e., weight of an average-sized individual within the population) by estimates of ungulate densities. Ungulate densities were estimated during the dry season of 2014 using distance-based line transect sampling, based on repeatedly walking 38 random transects (2–3 km in length) that were established in the core zone of SWS (see Rostro-García et al. 2018 for methodological details). Estimated densities (individuals/km^2^ ± *SE*) were 2.3 ± 0.5 for banteng, 2.1 ± 0.3 for muntjac, and 6.5 ± 1.9 for wild pig (Rostro-García et al. 2018). We assumed ungulate densities were similar in 2013, and therefore used one density estimate for both years of our study. We used adult female weights of 20 kg for muntjac, 75 kg for wild pig, and 600 kg for banteng, which were based on lower weight given for each species by Francis (2008).

To assess seasonal differences in diet, we divided the dry season into the cool-dry season (November – February) and hot-dry season (March – May) to parallel major changes in temperature and precipitation. Our study site became inaccessible during the main rainy season (June – October) because of high water levels and impassable rivers, so we could not collect jackal scats for this season. The cool-dry season had the lowest average daily temperatures per month (20.9 – 22.5°C) and lowest average rainfall per month (3 – 85 mm), whereas the hot-dry season had the highest average daily temperatures (24.3 – 25.0°C) with increasing average daily rainfall per month (50 – 306 mm; climate data were from nearby Sen Monorom, Cambodia, 1982 – 2012; climate-data.org; accessed on 10 July 2019). For each season, scats were pooled across years to obtain minimum sample sizes (> 50 scats/season). We used chi-square contingency tables to determine whether there were differences in diets between the two seasons that samples were collected. Diet, prey selection, and niche breadth values were calculated for each season, as well as total (i.e., combining data from both seasons).

## Results

We captured, collared, and monitored six adult jackals (three males, three females) from four different groups from February 2013 to February 2016 (Table 1). All jackals were considered alphas because they were adult breeders belonging to mated pairs and they associated with natal dens during May and June. No betas (adult helpers from previous litter) were observed or photographed by camera traps within the home ranges of mated pairs. One female jackal (3-F) died about a month after capture near the western edge of our study site. Her mate (2-M) was monitored for another 2 months until the start of the rainy season, but was not found the following dry season (Table 1). Due to the short period of monitoring and low number of locations, both of these jackals were removed from all analyses (Table 1). The area that this mated pair had occupied in 2013 was occupied subsequently by another mated pair, starting in 2014 (4-F, 5-M). Overall, three GPS-collared jackals were monitored for 6 – 7 months, all during the last half of the dry season and most of the rainy season (Table 1). One of these jackals (4-F) was recaptured the following year, radiocollared, and monitored during the subsequent dry season, during which time she used the same home-range area. We therefore pooled both the GPS and radiotelemetry of 4-F to calculate a single home range, which was similar in size the home ranges of the other GPS-collared jackals. One radiocollared jackal (1-F) was monitored for two consecutive dry seasons, and initial analysis showed she used the same home-range area, locations therefore were pooled to calculate a single home range. Although some jackals were captured in different years, and monitored for different periods (Table 1), all jackals included in the analyses were monitored simultaneously during 2015. There were two confirmed deaths of jackals during the study: 3-F, which had an unknown cause of death near the western edge of our study site; and 1-F, which died in an illegal snare set by poachers near the eastern edge of our study site (Table 1).

**Table 1.** Summary of data on golden jackals (*Canis aureus*) captured and monitored in Srepok Wildlife Sanctuary, Eastern Plains Landscape, Cambodia, 2013–2016. Note that all jackals captured were classified as alphas (adult breeders). Home-range sizes were calculated using fixed kernel density estimate (KDE) and minimum convex polygon (MCP).

ID-sex	Body mass (kg)	Collar type	No. of locations	Period monitored	KDE (km^2^)		MCP (km^2^)		Fate
					95%	50%	95%	50%	
1-F^a^	8.0	Radio	142	6 February 2013 to 15 February 2016	50.7	9.7	36.7	6.9	Died (humans)
2-M^b^		Radio	35	16 March 2013 to 8 May 2013					Unknown
3-F^b^	7.0	Radio	5	18 March 2013 to 12 April 2013					Died (unknown)
4-F^c^	9.7	GPS/Radio	204	26 January 2014 to 26 February 2016	46.7	6.8	34.9	5.2	Unknown
5-M^d^	8.0	GPS	737	13 March 2015 to 2 September 2015	40.1	9.0	38.9	7.9	Alive
6-M^e^	9.6	GPS	833	19 March 2015 to 15 October 2015	50.8	12.2	47.0	12.7	Alive

^a^Data from the first two consecutive dry seasons were used in the home-range analysis; died in an illegal snare set by poachers.

^b^2-M and 3-F were a mated pair; fate of 2-M was unknown, but possible dispersal from study site after his mate died.

^c^Mated pair with 5-M; 4-F was GPS-collared in 2014, then recaptured in 2015 and radiocollared; fate was unknown because contact was lost midway through the dry season of 2016.

^d^Mated pair with 4-F; 5-M was alive up until GPS collar fell off; ultimate fate was unknown.

^e^Alive up until GPS collar fell off; ultimate fate was unknown.

**Fig. 3.**
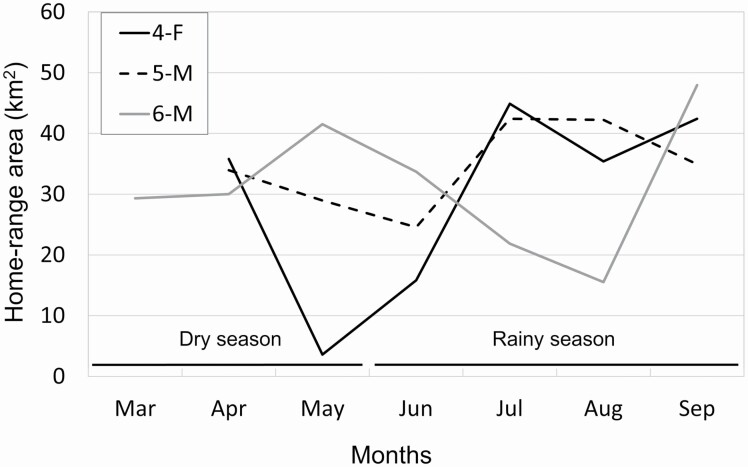
Monthly home-range sizes (95% fixed kernel density estimates) of three GPS-collared golden jackals (two males, one female) in Srepok Wildlife Sanctuary, Cambodia, 2014–2015. Note that 4-F and 5-M were a mated pair.

The mean (± *SE*) home-range size for jackals (*n* = 4) was 47.1 ± 2.5 km^2^ using 95% KDE, and 39.6 ± 2.7 km^2^ using 95% MCP (Table 1; Fig. 2). The mean (± *SE*) core-area size for jackals was 9.1 ± 1.1 km^2^ using 50% KDE, and 8.2 ± 1.6 km^2^ using 50% MCP (Table 1; Fig. 2). Home-range sizes were similar between the radiocollared jackal (1-F) and the GPS-collared jackals, even though the radiocollared jackal (1-F) was monitored for a longer period (Table 1). Both collar types therefore produced similar results, probably because of the relatively large number of locations obtained per jackal. Monthly home-range sizes (95% KDE) for the three GPS-collared jackals ranged from 4 to 45 km^2^ for 4-F (28 – 30 locations/month), 26–42 km^2^ for 5-M (90–129 locations/month), and 16 – 48 km^2^ for 6-M (98 – 127 locations/month; Fig. 3). Because of differences in GPS-collar programming, 4-F had fewer locations per month than the other two jackals. However, the monthly home ranges of 4-F were similar in size to her mate (5-M), except for the birthing and pup-rearing seasons in May (4 km^2^) and June (16 km^2^; Fig. 3); we therefore assumed the estimated monthly home ranges of 4-F were accurate despite the lower number of locations. There was no overlap between the two jackals with adjacent home ranges (1-F, 6-M; Fig. 2). The overlap of the mated pair (4-F, 5-M) was 1.41 using UDOI 95, and 90.3% using the percent area overlap (based on 95% MCP; Fig. 2), whereas overlap of their core areas was 0.32 using UDOI 50, and 64.4% using the percent area overlap (based on 50% MCP; Fig. 2).

The estimated pre-whelping density of jackals in our study was 0.01 jackal/km^2^. This was based on eight resident jackals from four mated pairs occupying the 800-km^2^ intensive study area (Fig. 2). Although we collared and simultaneously monitored jackals from three mated pairs occupying three distinct areas, our observations of jackal sign (i.e., prints and scats), along with photographs from camera-trap sites, confirmed that a fourth mated pair of jackals occurred within our study. The estimated home range of the fourth mated pair was situated between the home ranges of the collared jackals (Fig. 2). All other camera-trap sites with multiple photographs of jackals occurred only within the home ranges of our collared jackals (Fig. 2). Photographs from the camera-trap surveys showed that jackals and large carnivores occupied different areas within the study site: the four mated pairs of jackals occupied the northern and western part of our study site, whereas leopards and dholes were recorded only in the south central part of the study area (Fig. 2).

Although locations obtained by radiocollars were less accurate than the locations obtained by GPS collars, results from both collar types showed the same patterns in resource selection; locations from both collar types therefore were used in the analysis. We eliminated DDF because it had a strong correlation with dense forest, and used the remaining variables to construct our resource selection model. Jackals had strong avoidance of dense forests, and strong selection for dirt roads (Table 2). In addition, jackals had a significant avoidance of streams (Table 2). Results from blocked cross-validation indicated that our model had strong predictive power (*r*_s_ = 0.903; *P* < 0.0001).

**Table 2.** Summary of the results of the resource selection model for collared jackals in eastern Cambodia, 2013–2016. Shown are selection coefficients (β), standard error (*SE*), 95% confidence intervals (LCL = lower confidence limit; UCL = upper confidence limit), *z*-scores, and *P*-values.

Variable	β	*SE*	95% LCL	95% UPL	*z*	*P*
Dense forests	0.470	0.116	0.242	0.698	4.053	< 0.0001
Roads	−0.362	0.092	−0.542	−0.182	−3.940	< 0.0001
Streams	0.141	0.062	0.019	0.263	2.720	0.023

We used 147 scats of jackals to determine their diet and prey selection, which were collected during the dry seasons of 2013 (*n* = 89) and 2014 (*n* = 58). The mean (± *SE*) scat diameter was 2.4 ± 0.1 cm (range = 1.8 – 3.2 cm; *n* = 113). Most jackal scats were found singly (77.6%), whereas the others were found in groups of two or three on the same shrub or tuft of grass. Relatively few scats contained only one prey item (14.4%), whereas remaining scats contained two (38.4%), three (27.4%), four (17.1%), or five (2.7%) prey items. Overall, we identified the remains of at least 16 prey items in jackal scats, including three ungulate species (Table 3). Processional termites comprised 25.6% of biomass consumed, followed by wild pigs (20.3%), muntjac (20.1%), civets (17.2%), small rodents (6.0%), and hares (4.6%; Table 3). No other prey items comprised > 4% of biomass consumed. Processional termites were by far the most frequently consumed prey item, being found in 76.9% of all scats (Table 3). Processional termites also dominated the content of scats, as 52.4% of all scats were comprised of > 50% termites, and 40.1% of all scats were comprised of > 75% termites.

**Table 3.** Seasonal and total diet composition expressed as percentage of ingested biomass (Bio), percentage of scat volume (Vol), and frequency of occurrence (Occ) of golden jackals in Srepok Wildlife Sanctuary, eastern Cambodia, 2013 – 2014 (*n* = number of scats analyzed). Dietary niche breadth (*B*) is given based on biomass consumed.

	Cool-dry (*n* = 69)			Hot-dry (*n* = 78)			Total (*n* = 147)		
Prey category^a^	Bio	Vol	Occ	Bio	Vol	Occ	Bio	Vol	Occ
Ungulate	39.3	14.8	30.4	41.9	11.0	33.3	40.7	12.8	32.0
Northern red muntjac (*Muntiacus vaginalis*): 20–28 kg	21.6	6.6	8.7	18.7	4.4	9.0	20.1	5.4	8.8
Wild pig (*Sus scrofa*): 75–200 kg	17.7	8.2	21.7	22.8	6.6	24.4	20.3	7.3	23.1
Large cervid^b^	0.0	0.0	0.0	0.4	0.1	1.3	0.2	< 0.1	0.7
Civet^c^	21.8	14.7	21.7	12.9	8.8	17.9	17.2	11.6	19.7
Burmese hare (*Lepus peguensis*): 2–3 kg^d^	8.1	4.8	7.2	1.3	1.5	2.6	4.6	3.1	4.8
Malayan porcupine (*Hystrix brachyura*): 8 kg^e^	0.0	0.0	0.0	0.2	0.1	1.3	0.1	0.1	0.7
Small rodent: < 1 kg	6.8	14.9	26.1	5.3	9.1	17.9	6.0	11.8	21.8
Bird: < 1 kg	1.7	2.2	7.2	4.8	5.1	21.8	3.3	3.7	15.0
Reptile: < 0.5 kg	0.7	2.0	18.8	2.1	4.4	19.2	1.4	3.3	19.0
Small lizard	0.3	0.9	5.6	0.6	1.2	3.8	0.4	1.1	4.8
Small snake	0.1	0.1	2.9	0.1	0.1	3.8	0.1	0.1	2.7
Small tortoise	0.0	0.0	0.0	< 0.1	0.1	1.3	< 0.1	< 0.1	0.7
Unidentifiable reptile	0.3	0.9	10.1	1.4	3.0	11.5	0.9	2.0	10.9
Arthropod: < 0.01 kg	21.2	46.2	76.8	31.5	57.8	84.6	26.6	52.4	81.0
Processional termite (*Hospitalitermes* spp.)	20.4	41.2	72.4	30.4	53.2	80.8	25.6	47.6	76.9
Beetle (Coleoptera)	0.1	0.3	5.8	0.7	2.4	12.8	0.4	1.5	9.5
Grasshopper (Orthoptera)	< 0.1	0.1	1.4	0.0	0.0	0.0	< 0.1	< 0.1	0.7
Fresh-water crab (Potamidae)	0.8	4.6	20.3	0.4	2.2	16.7	0.6	3.3	18.4
Egg shell	< 0.1	0.1	1.4	0.0	0.0	0.0	< 0.1	< 0.1	0.7
Seeds	0.3	0.2	2.9	0.0	0.0	0.0	0.1	0.1	1.4
Unidentifiable		0.1	1.4		2.2	6.4		1.2	4.1
Niche breadth (*B*)	5.59			4.95			5.43		

^a^Body weights of mammalian species were taken from Francis (2008), unless otherwise noted.

^b^Sambar (*Rusa unicolor*; 180–260 kg) or Eld’s deer (*Rucervus eldii*; 95–150 kg) remains comprised 5% of one scat during the hot-dry season.

^c^Asian palm civet (*Paradoxurus hermaphroditus*; 2–3 kg), small Indian civet (*Viverricula indica*; 2–4 kg), large Indian civet (*Viverra zibetha*; 8–9 kg), or large-spotted civet (*Viverra megaspila*; 8–9 kg).

^d^Body weight taken from Nowak (1999).

^e^Body weight taken from Flux and Angermann (1990).

**Fig. 4.**
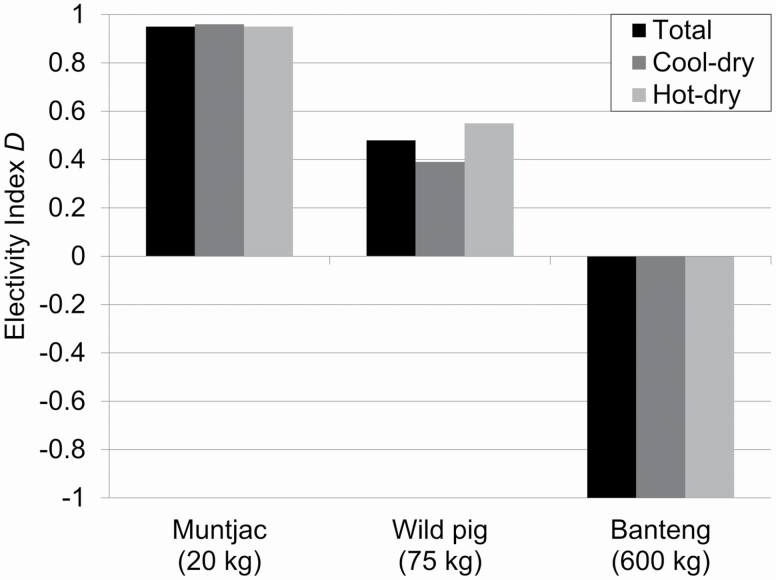
Jacobs’ (1974) electivity index (*D*) of ungulates based on percent biomass consumed by golden jackals in Srepok Wildlife Sanctuary, Cambodia. Body mass of adult female ungulates is given, taken as the lower range of body mass reported by Francis (2008).

Diets did not differ between the cool-dry and hot-dry seasons (χ ^2^ = 10.237, *P* = 0.332). Niche breadth values were similar in both seasons and overall (Table 3). The dietary overlap (*R*_0_) was 0.61 between jackals and dholes, and 0.56 between jackals and leopards. The percentage of ungulate biomass available in the core zone in SWS was 71% banteng, 26% wild pig, and 2% muntjac. The banteng is a DDF specialist, whereas the wild pig and muntjac are habitat generalists (Gray et al. 2013); therefore, all three ungulates occur in DDF and were available to jackals. The total biomass of ungulates consumed by jackals did not reflect the biomass available, because jackals showed a strong selection for muntjac (*D* = 0.95) and a moderate selection for wild pig (*D* = 0.48), but a complete avoidance of banteng (*D* = −1.00, Fig. 4). Prey selection values were similar in both seasons and overall (Fig. 4).

## Discussion

The home-range sizes of jackals in our study were considerably larger than those reported in previous studies. We confirmed that all collared jackals were from mated pairs that had natal dens. We therefore are confident that these home ranges represent resident alphas and not betas or transients. Not only are the home-range sizes in our study the largest reported for resident breeding jackals in Eurasia, they also are the largest reported for adult residents of any mesocanid species in Africa (Moehlman and Hayssen 2018; Kamler et al. 2019). Our results supported our prediction that jackals in SWS would have relatively large home ranges because our study occurred in a natural area. The main reason for the unusually large home ranges of jackals in SWS, even compared to other natural areas, likely was due to the extreme seasonal changes in herbaceous cover and water availability that occurred on our study site, and the subsequent influence this had on prey availability. The DDF dominated our study site, and its grassy understory annually burns during the dry season after the trees lose their leaves; thereafter, the landscape remains barren of leaves and grasses for several months until the rainy season starts (McShea and Davies 2011). In addition, small rivers within the study site stop flowing or dry up completely, and > 90% of the waterholes dry up (Pin et al. 2020). The effect of decreased water availability on home ranges of jackals is unknown, although coyote home ranges in desert environments were not influenced by free-standing water (Kluever and Gese 2016), so the same may be true of jackals. Instead, the extreme change in herbaceous cover and water availability during the dry season appeared to cause a dramatic decrease in numbers of potential prey, particularly small vertebrates, thereby severely limiting the food available to jackals. For example, after dry-season fires in dry dipterocarp forests in Thailand, the biomass of small mammals was found to decrease 76% (Walker and Rabinowitz 1992) and relative abundance of ground squirrels was found to decrease about 70% (Kobayashi et al. 2017). Numbers of amphibians and small reptiles also decrease in DDF during the dry season (Zug 2011), primarily because most species go into torpor in burrows during the dry season. Thus, jackal home ranges and land requirements probably have to be unusually large in DDF to incorporate the extreme seasonal fluctuations in availability of small vertebrate prey.

The unusually large home ranges, which were mutually exclusive between different mated pairs, contributed to an extremely low density of jackals (0.01 jackal/km^2^) in SWS. Although we had predicted that jackals would have a relatively low density in SWS because it was a natural area, we did not expect densities to be several orders of magnitude lower than that reported in India (1–17 jackal/km^2^—Jhala and Moehlman 2004; Prerna et al. 2015; Singh et al. 2016). Jackal densities in our study also were considerably lower than estimated densities of jackal groups in Europe (Šálek et al. 2014; Lanszki et al. 2015; Trbojević et al. 2018; see Singh et al. 2016 for summary of jackal densities).

The monthly home ranges calculated for three jackals also were relatively large, typically 20–50 km^2^, during both dry and rainy seasons (Fig. 3). There did not appear to be any strong seasonal pattern in home-range sizes, with the exception of 4-F. The home-range size of this female was smallest in May (4 km^2^), during which time she gave birth to pups (Fig. 3). Her monthly home range increased to 16 km^2^ in June, when pups were 4–8 weeks old, then returned to a size similar to that of her mate’s (5-M) in July and thereafter (35–44 km^2^; Fig. 3); this indicated her smaller home ranges in May and June were influenced by the birthing and pup-rearing periods, respectively. Reasons for the different sized monthly ranges of 5-M and 6-M were unknown, although it could be have been due to differences in food diversity and abundance, given that these jackals had home-range boundaries that were about 15 km apart. The lack of seasonal differences in home-range sizes, despite the seasonal changes in small vertebrate prey, suggests that jackals maintained home ranges year-around that were large enough to incorporate seasonal decreases in prey, which is consistent with the resource dispersion hypothesis (Macdonald 1983).

Jackal core areas never occurred in areas where leopards and dholes were photographed by camera traps, and both large carnivores only were recorded near the edge, or outside, of jackal home ranges (Fig. 2). There was no apparent difference in habitat types between areas dominated by jackals and those dominated by large carnivores, suggesting jackals were spatially displaced in some areas by large carnivores, thereby further contributing to the low density of jackals in SWS. Spatial partitioning between dominant carnivores and subordinate canids is a common mechanism of coexistence, and could be due to either behavioral avoidance (Kamler et al. 2012, 2013) or excessive predation on subordinate canids in areas occupied by dominant carnivores (Kamler et al. 2003). The results of resource selection showed that jackals avoided dense forests and streams, which did not support our prediction that jackals would use habitats in proportion to availability. The avoidance of dense forests and streams by jackals might have been a strategy to avoid leopards, which prefer these habitats in SWS (S. Rostro-García, University of Oxford, pers. comm.). Jackals had a strong selection for dirt roads, including a public road near the western edge of our study area, as well as smaller roads within the northern part of our study area that were used almost daily by rangers on motorcycles. The selection for roads also might have been a strategy to avoid leopards and dholes, because both of these large carnivores avoid areas of high human activity within protected areas (Srivathsa et al. 2014; Ngoprasert et al. 2017). Previous studies have shown that roads can function as a refuge for subordinate canids to avoid dominant carnivores (Sargeant and Allen 1989; Kamler et al. 2003). Alternatively, roads may have been preferred by jackals to better facilitate their movements while traversing their unusually large home ranges. Because camera traps were placed along roads, and jackals selected for roads, it was highly unlikely that the camera traps failed to detect any other mated pairs of jackals within our study area.

No beta jackals were captured or observed in any of the home ranges in SWS, which suggests that low food resources during the dry season were affecting group size and social organization. Previous research showed that beta jackals remain philopatric if there are adequate food resources within their natal range, and that mated pairs of jackals can live with up to three betas within their home ranges (Kamler et al. 2019), and possibly more if food resources are super abundant (Macdonald 1979). According to the resource dispersion hypothesis (Macdonald 1983), low patch richness of prey, such as termites or civets, also might have contributed to low group sizes of jackals. The lack of beta jackals within the home ranges of alphas also contributed to the low density of jackals in SWS.

The most important prey item of jackals in SWS during the dry season was processional termites, based on biomass consumed, percent volume, and frequency of occurrence (Table 3), which was unexpected. Processional termites were found in 77% of all scats, and termites comprised a majority of most scats, indicating this prey item was regularly consumed in large amounts by jackals during the dry season. No other *Canis* species has been found to consume such a high amount of small insects, or to have a small insect as the most important prey item. In fact, the relatively high amount of termites in the jackal diet in SWS is more similar to the amount of termites in the diet of the myrmecophagus bat-eared fox (*Otocyon megalotis*) in Africa (Klare et al. 2011b), than to diets of other jackal populations. Nevertheless, energetic models have shown that carnivores, including jackals, weighing up to 21.5 kg can theoretically sustain themselves on small invertebrates (Carbone et al. 1999). Reasons for the high consumption of termites by jackals in SWS were not clear, but we suspect it was due to low amounts of alternative prey during the dry season. It is likely that major decreases in availability of small vertebrate prey in DDF after dry-season fires caused processional termites to become one of the most important food items of jackals during the dry season. It is noteworthy that processional termites, which forage aboveground in dense wide columns, are native to Asia, but not Europe or Africa; therefore, only jackals in Asia have the potential to lick up large amounts of termites when foraging. For example, up to 500,000 individuals can be involved in the nightly foraging excursion of a single group of processional termites (Collins 1979). In contrast, harvester termites (*Hodotermes mossambicus*) in Africa travel aboveground in single files; thus, they presumably are less efficient for jackals to lick up in large quantities, which helps explain why jackals in Africa consume only negligible quantities of termites, even in habitats where termites are common (Klare et al. 2010). We conclude that the dense wide columns of processional termites are relatively efficient for jackals to lick up and consume in large quantities, thereby providing an important source of protein for jackals during the dry season in DDF, especially when larger prey items become less available. Interestingly, concurrent dietary studies in SWS using DNA-confirmed scats showed that leopards, dholes, jungle cats, and leopard cats did not consume processional termites beyond negligible amounts (Rostro-García et al. 2018, In press; Kamler et al. 2020d), indicating jackals were the only canid or felid species to take advantage of this food resource during the dry season.

Contrary to our prediction, small rodents and hares were not major components of the jackal diet during the dry season in SWS. Our result differs from those of several studies that showed the main prey of jackals to be small mammals (Hayward et al. 2017; Supplementary Data SD1). The relatively low amount of small mammals in the jackal diet in SWS likely was related to the dry-season fires in DDF forests, which have been shown to reduce the numbers and biomass of small mammals (Walker and Rabinowitz 1992; Kobayashi et al. 2017). Overall, there were no seasonal differences in diets between the cool-dry and hot-dry seasons, indicating low prey availability was relatively constant across the entire dry season.

Ungulates comprised a relatively high proportion (41%) of the jackal diet during the dry season in SWS, with muntjac and wild pig being consumed in equal amounts based on biomass consumed. The relatively high consumption of ungulates could have been in response to the large decrease in availability of small vertebrates during the dry season. Jackals were not random in their consumption of ungulates, because muntjac were selectively consumed over other ungulates during both seasons, based on biomass available. Scat analysis alone does not allow for reliable differentiation between killed and scavenged prey. However, we conclude that nearly all consumption of ungulates by jackals on our study site was due to active hunting rather than scavenging for the following reasons: (1) body size of ungulates influenced the prey selection of jackals, because the smallest ungulate was selectively consumed over a medium-sized ungulate (i.e., wild pig), whereas the largest ungulate (banteng) was not consumed at all; (2) the strong preference for muntjac was not related to a high prey density of this species, because the density of wild pigs was three times higher than that of muntjac, and the density of banteng was similar to muntjac; (3) if ungulates had been scavenged, then consumption would have been similar to biomass available assuming ungulates had similar rates of natural mortality; (4) the strong selection for muntjac and avoidance of banteng by jackals in SWS likely was not the result of scavenging from kills of larger carnivores based on our results of dietary overlap among carnivores; (5) previous studies showed that jackals and other mesocanids have a consistent and strong selection for small- and medium-sized ungulate species that hide their young after birth (Klare et al. 2010; Hayward et al. 2017), and muntjac were the only small ungulate hider species on our study site.

There were no realistic circumstances where scavenging on carcasses would have explained our dietary results for jackals. For example, although we did not determine mortality rates among ungulate species, it would have been highly unlikely that muntjac would consistently be dying at 10 times the rate of banteng across both seasons and years that were studied, particularly given that we never found ungulate carcasses on our study site. Even though some illegal snaring probably occurred in the core zone of SWS during our study (Rostro-García et al. 2018), snaring in the region is indiscriminate (Gray et al. 2017) and thus any potential ungulate remains from snaring would have been representative of available ungulate biomass. Leopards and dholes preyed almost exclusively on ungulates in SWS, with banteng comprising 19% of dhole diet (Kamler et al. 2020d) and 42% of the leopard diet (Rostro-García et al. 2018). However, we did not detect banteng in the scats of jackals, indicating that jackals in SWS were not regularly scavenging from ungulates killed by dholes and leopards, at least during the dry season, which did not support our prediction.

Previous research on black-backed jackals and African wolves showed that these species were major predators of fawns of small- and medium-sized ungulate species that hide their young (Lamprecht 1978; Moehlman and Hayssen 2018), and that fawns of these ungulate species were preferentially hunted over fawns of other ungulate species (Klare et al. 2010; Hayward et al. 2017). Our results on golden jackals appear consistent with the above studies, because muntjac, which breed year-around (Francis 2008), are a small hider species that had a consistently high selection by jackals. Some previous studies on golden jackals support our conclusion, because jackals were found to prey of fawns of chital, blackbuck, and nilgai (Eisenberg and Lockhart 1972; Supplementary Data SD1), and newborn cattle calves (Yom-Tov et al. 1995), all of which are hider species. In addition, we found adult-sized hooves of muntjac in two jackal scats, indicating jackals in SWS also were consuming adult muntjac, which is consistent with previous reports of black-backed jackals, African wolves, and coyotes, preying on adults of small-sized ungulates (Bekoff and Gese 2003; Kamler et al. 2010; Klare et al. 2010; Moehlman and Hayssen 2018).

Jackals in SWS also consumed wild pig, although this prey species was not as highly selected as were muntjac. The relatively high consumption of wild pig (20% of diet) was somewhat surprising, given that wild pigs are not a hider species, and are gregarious and relatively aggressive; as a result, jackals tend to avoid wild pig (Lanszki and Heltai 2010; Hayward et al. 2017). Although subadult and adult wild pigs may have been too large and aggressive for jackals to prey upon, piglets may have been vulnerable to jackal predation. Firstly, wild pigs have relatively large litters (up to 12 young/litter—Nowak 1999); consequently, some piglets might become susceptible to predation if they become sick or wander too far from the group. Secondly, we found piglet hooves, but no adult hooves, in two jackal scats, indicating at least some piglets were consumed by jackals. Thirdly, research in Hungary showed that when hunter-killed carrion was removed from a game management area, jackals increased their consumption of piglets and young wild pigs, presumably via predation (Lanszki et al. 2018). We suspect that because of the relatively high density of wild pigs in SWS (6.5 individuals/km^2^), jackals took advantage of this relatively abundant food source by opportunistically preying on piglets and young wild pig, especially when the seasonal availability of other prey species decreased.

Civets comprised 22% of the jackal diet in the cool-dry season, and 17% of the jackal diet for the entire dry season, which was similar to the amounts of muntjac and wild pig in their diets. This result was surprising, given that small carnivores were never found to be common prey items in previous dietary studies of jackals. In fact, the amount of civets in the jackal diet in SWS was the highest ever reported for small carnivores in a jackal diet. Only two previous studies found civets in jackal diets, one in India (Khan et al. 2017) and another in Pakistan (Shabbir et al. 2013), although in both instances they comprised relatively small amounts of the jackal diets (1% and 9%, respectively). Other small carnivores have been detected in low amounts in previous studies of jackal diets, including domestic dogs and domestic cats (Supplementary Data SD1), indicating jackals can regularly prey on smaller carnivores. Reasons for the high consumption of civets by jackals in SWS were not clear, but we suspect it was due to the decrease in numbers of small vertebrate prey species during the dry season, thereby causing civets to become an important buffer food during the dry season. Four species of civets are relatively common in DDF in SWS, including one species (large-spotted civet) that uses DDF exclusively (Gray et al. 2010). Consequently, civets, which are primarily terrestrial (Francis 2008), were widely available as prey to jackals in the DDF, even during the dry season. Unfortunately, we did not determine abundance of civets, small mammals, or termites, on our study site; we therefore could not determine the dietary preference of these prey groups by jackals.

A limitation of our study was that we could not collect jackal scats during the rainy season; we therefore could not compare diets between the rainy and dry seasons. Given the relatively harsh conditions during the dry season in DDF, including annual burning of herbaceous cover and limited water availability, it is possible that jackal diets are quite different during the rainy season. For example, the abundance of small mammals and herpetofauna both increase during the rainy season in DDF, therefore, these prey categories might increase in importance in the jackal diet during the rainy season. Conversely, the amount of processional termites in the jackal diets might decrease during the rainy season as other food resources become available. Nevertheless, the dry season likely is the most limiting period for populations of jackals in the DDF, determining their diets during the dry season therefore was important for understanding their ecological needs.

Overall, our results show that the jackal is a very adaptable and opportunistic species that exhibits extreme intraspecific flexibility with respect to home-range size, social organization, diet, and ecological role as predator or scavenger. In fact, the intraspecific variation exhibited by jackals might be greater than the variation exhibited by many distantly related canid species (Macdonald and Sillero-Zubiri 2004). Jackals in the DDF had the largest home ranges, and presumably largest land requirements, of any previously studied jackal population, probably because of the decrease in numbers of small vertebrates during the harsh dry season, and overall lack of human-generated foods. The unusually large home ranges, lack of betas, and potential spatial avoidance of large carnivores, contributed to an extremely low density of jackals. Nevertheless, jackals persisted in SWS and adapted to the harsh dry season by consuming large amounts of processional termites, as well as civets, at higher quantities than had previously been reported for jackals in other habitats. In addition, in the absence of human-generated carrion, jackals appeared to actively prey on small ungulates and young of medium-sized ungulates. Yet jackals apparently did not scavenge from ungulates killed by larger carnivores, probably to avoid being preyed upon themselves. Future research should investigate the social organization of jackals in DDF in more detail, as well as their diets during the rainy season, their dietary preferences among all prey groups, and their interspecific relationships with larger carnivores. This would result in a more complete understanding of the behavior, resource use, and ecological needs of this species in an extreme environment near the edge of their distribution.

## Supplementary Data

Supplementary data are available at *Journal of Mammalogy* online.

Supplementary Data SD1.–Summary of 49 dietary studies of golden jackals in Europe and Asia.

gyab014_suppl_Supplementary_Material
