# Supplementary material for: Home range, habitat selection, density, and diet of golden jackals in the Eastern Plains Landscape, Cambodia
Source: J Mammal. 2021 Mar 20;102(2):636–50. doi: 10.1093/jmammal/gyab014 (PMC8491366; doi:10.1093/jmammal/gyab014)
Supplement: gyab014_suppl_Supplementary_Material [file gyab014_suppl_supplementary_material.docx]

# Supplementary Data S1 – Summary of 48 dietary studies of golden jackals in Europe and Asia*

| Country | Habitat | Human-based food available | Main prey items | Other important prey items | Reference |
| --- | --- | --- | --- | --- | --- |
| EUROPE |  |  |  |  |  |
| Azerbaijan | Human-altered: Ismailli District | Carrion | Bird, herpetofauna, fruit, insects | Carrion | Rukovskii 1953, as cited in Heptner and Naumov 1998 |
| Azerbaijan | Human-altered: Kurdamir District | Carrion | Nutria, small rodent, bird | Insect | Pavlov 1953, as cited in Heptner and Naumov 1998 |
| Bulgaria | Human-altered: farmland | Crops | Small rodent, hare | Fruit  Minor: crops | Markov and Lanszki 2012 |
| Bulgaria | Human-altered: Farmland, forest & plantations | Livestock carrion, poultry carrion, wild ungulate carrion, dog, cat, garbage | Livestock carrion, poultry carrion, wild ungulate carrion, dog, garbage, small rodent | Hare, crops, cat  Minor: Red fox, European badger | Raichev et al. 2013 |
| Bulgaria | Human-altered: Farmland, forest & plantations | Livestock carrion, poultry carrion, wild ungulate carrion, dog, cat, garbage | Livestock carrion, wild ungulate carrion | Small rodents, garbage, fruit, European badger, red fox | Tsunoda et al. 2017 |
| Bulgaria | Human-altered: forest & plantations | Wild ungulate carrion, dog, cat, garbage | Small rodent, fruit | Small carnivores  Minor: garbage | Tsunoda et al. 2019 |
| Croatia | Human-altered: farmland & forest | Sheep, dog, crops | Fruit, sheep, insect, wild pig carrion | Hare  Minor: dog | Radović and Kovačić 2010 |
| Croatia | Human-altered: eastern Croatia | Livestock carrion, Wild ungulate waste/carrion | Livestock carrion, Wild ungulate waste/carrion | Small mammal, fruit | Bošković et al. 2013 |
| Georgia | Human-altered: near Gagra, Abkhazia | Livestock carrion | Bird, small rodent, nutria, livestock carrion | Fish carrion | Pavlov 1953, as cited in Heptner and Naumov 1998 |
| Greece | Human-altered: farmland & marsh | Goat, sheep, dog, cat, crops | Goat carrion, bird | Wild pig carrion  Minor: dog, cat, crops, least weasel | Lanszki et al. 2009 |
| Greece | Human-altered: farmland & marsh | Goat, sheep, dog, cat, crops | Goat carrion | Wild pig carrion  Minor: dog, cat, crops | Lanszki et al. 2010 |
| Greece | Human-altered: farmland | Sheep, pig, dog, cat, rabbit, crops | Sheep, dog, rabbit, cat, pig | Birds, stone marten, crops | Giannatos et al. 2010 |
| Hungary | Human-altered: farmland | Wild ungulate carrion, crops | Small rodent, wild ungulate carrion | Minor: crops, stone marten | Lanszki and Heltai 2002 |
| Hungary | Human-altered: farmland & forest | Wild ungulate carrion, cattle, pig, cats | Small rodent, wild pig piglet, cattle carrion, wild ungulate carrion | Minor: least weasel, red fox, cat | Lanszki et al. 2006 |
| Hungary | Human-altered: farmland & forest | Poultry, dog, cat, crops | Small rodents, fruit | Wild pig carrion  Minor: dog, cat, poultry, crops | Lanszki et al. 2010 |
| Hungary | Human-altered: farmland & forest |  |  |  | Lanszki and Heltai 2010 |
| Hungary | Human-altered: farmland & forest | Wild ungulate waste/carrion, dogs, crops | Wild ungulate waste/carrion | European badger  Minor: dog | Lanszki et al. 2015 |
| Hungary | Human-altered: farmland & forest | Wild ungulate waste/carrion, dogs, crops | Small rodents, wild ungulate carrion, | Fruits  Minor: Wild pig piglet, European badger, cat, crops | Lanszki et al. 2016 |
| Hungary | Human-altered: farmland & forest | Wild ungulate carrion, dog, cat, crops | Wild ungulate waste/carrion | Wild piglets, insects, plants  Minor: European badger | Lanszki et al. 2018 |
| Serbia | Human-altered: regional | Pig, goat, sheep, poultry, dog | Pig carrion | Goat carrion, sheep carrion, roe deer carrion, dog | Ćirović et al. 2014 |
| Serbia | Human-altered: farmland & forest | Livestock, poultry, dog, cat, wild ungulate carrion, garbage | Livestock carrion, small rodents, poultry carrion | Wild ungulate carrion  Minor: dog, cat, small carnivores | Aleksandra and Duško 2015 |
| ASIA |  |  |  |  |  |
| Bangladesh | Human-altered: farmland | Livestock, poultry, crops, garbage | Livestock carrion, poultry | Birds, fruit, crops | Poché et al. 1987 |
| Bangladesh | Human-altered: farmland | Livestock, poultry, garbage | Rodents, birds | Cattle carrion, crops, garbage, insects | Jaeger et al. 2007 |
| Cambodia | Natural: Srepok WS | None | Processional termites, wild pig, muntjac, civets | Small rodent, hare | This study |
| India | Natural: Eravikulam NP | Dogs | Small rodent, reptile | Insect, wild ungulate carrion, bird | Rice 1986 |
| India | Natural: Keoladeo NP | Cattle | Small rodent, bird, fruit | Chital, insects | Sankar 1988 |
| India | Natural: Keoladeo NP | Cattle, garbage | Nilgai fawn, livestock carrion, small rodent | Chital fawn  Minor: mongoose, garbage | Singh et al. 2016 |
| India | Natural: Sariska TR | Cattle, goat, garbage | Small rodent, bird | Reptiles, insect, vegetation, wild ungulate carrion, garbage | Mukherjee et al. 2004 |
| India | Natural: Sariska TR | Cattle, goat | Chital carrion, cattle carrion | Sambar & nilgai carrion | Gupta 2011 |
| India | Natural: Sariska TR | Cattle, goat | Small rodent, cattle carrion, chital fawn | Fruit, bird, insect, sambar & nilgai carrion | Chourasia et al. 2012 |
| India | Natural: Velavadar NP | Cattle, domestic buffalo, village garbage | Blackbuck fawn, cattle carrion | Wild ungulate carrion, vegetation, hare, buffalo carrion | Aiyadurai and Jhala 2006 |
| India | Natural: Pench TR | Cattle | Small rodents, chital fawn | Hare, reptile  Minor: cattle | Majumder et al. 2011 |
| India | Natural: Kanha TR | Cattle carrion, village garbage, grain crops | Rodent, fruit, reptile, bird, fish, garbage, grain crops | Cattle carrion, wild ungulate carrion | Schaller 1967 |
| India | Natural: Van Vihar NP | Provisioning of wild ungulate carrion, garbage | Chital fawn, nilgai fawn, fruit, garbage | Small rodent, wild ungulate carrion | Prerna et al. 2015 |
| India | Natural: Gir NP | Cattle, domestic buffalo, garbage | Buffalo carrion, chital fawn, hare | Sambar, small rodent, cattle carrion | Alam et al. 2015 |
| India | Human-altered & natural: Patna Bird Sanctuary | Cattle | Birds, small rodent, fruits | Minor: hare, cattle carrion, palm civet | Khan et al. 2017 |
| India | Natural: Pirotan Island (marine NP) | Garbage? | Crabs | Fish | Ramkumaran et al. 2017 |
| India | Human-altered & natural: Himalayan region, NW India | Fruit crop, grain crop, livestock carrion | Rodent, reptile, land crab | Minor: insect, fruit crop, grain crop, wild ungulate carrion | Schaller 1977 |
| Israel | Natural: nature reserve | Poultry carrion, livestock carrion, garbage | Poultry carrion, garbage | Fruit | Macdonald 1979 |
| Israel | Human-altered: vinyards and orchards | Poultry, dog, cat | Poultry carrion | Minor: dog, cat, cattle carrion, crops | Lanszki et al. 2010 |
| Israel | Human-altered: Britannia Park & farmland | Cattle, goat, sheep, camel, garbage | Carrion of cattle, goat, sheep and camel, wild ungulate carrion | Minor: Poultry, garbage | Borkowski et al. 2011 |
| Pakistan | Human-altered: farmland (Punjab) | Livestock, crops | Small rodent, bird | Minor: reptile, hare | Khan and Beg 1986 |
| Pakistan | Human-altered: livestock pastures | Cattle, sheep, goat, poultry, garbage | Livestock carrion | Small rodents, poultry carrion, antelope carrion, fruit | Nadeem et al. 2012 |
| Pakistan | Natural: Margallah Hills NP | Cattle, goat, sheep, dog, fruit crop, garbage | Livestock carrion, wild pig carrion, garbage | Mongoose, crops  Minor: dog | Mahmood et al. 2013 |
| Pakistan | Natural: Chitral Gol NP & Tushi GR | Cattle, sheep, goat | Small rodent, birds, insects | Palm civet, sheep, hare, marmot | Shabbir et al. 2013 |
| Pakistan | Natural: Pir Lasura NP | Cattle, buffalo, sheep, goat, poultry, garbage | Poultry carrion, goat, garbage | Minor: Cattle carrion, macaque, wild pig carrion | Akrim et al. 2019 |
| Tajikistan | Human-altered: Near Amu Darya River | Fruit crop, carrion, garbage | Rodent, bird, insect, fruit crop, wild fruit, carrion, garbage, fish | Minor: European badger, marbled polecat | Chernyshev 1958, as cited in Heptner and Naumov 1998 |
| Uzbekistan | Natural: Aral Paygambar Reserve | Wild ungulate carrion | Insect, fruit, hare, rodent | Minor: wild ungulate carrion | Volozheninov 1972 |

* The golden jackal was once thought to occupy eastern and northern Africa, although those mesocanids are now classified as the African wolf (*C. lupaster* or *C. anthus*; Hoffman et al. 2018). Therefore, dietary studies of mesocanids identified as golden jackals in eastern or northern Africa were excluded from this table.

Literature Cited

Aiyadurai, A., and Y. V. Jhala. 2006. Foraging and habitat use by golden jackals (*Canis*

*aureus*) in the Bhal region, Gujarat, India. Journal of the Bombay Natural History Society 103:5-12.

Akrim, F., T. Mahmood, M. S. Nadeem, T. Dhendup, H. Fatima, and S. Andleeb. 2019. Diet

composition and niche overlap of two sympatric carnivores: Asiatic jackal *Canis aureus* and Kashmir hill fox *Vulpes vulpes griffithii*, inhabiting Pir Lasura National Park, northeastern Himalayan region, Pakistan. Wildlife Biology, In press.

Aleksandra, P., and Ć. Duško. 2015. Seasonal variation in diet of the golden jackal (Canis

aureus) in Serbia. Mammal Research 60:309-317.

Alam, M. S., J. A. Khan, C. H. Njoroge, S. Kumar, and R. L. Meena. 2015. Food preferences

of the golden jackal *Canis aureus* in the Gir National Park and Sanctuary, Gujarat, India. Journal of Threatened Taxa 7:6927-6933.

Borkowski, J., A. Zalewski, and R. Manor. 2011. Diet composition of golden jackals in

Israel. Annales Zoologici Fennici 48:108-118.

Bošković, I., et al. 2013. Dietary habits of the golden jackal (*Canis aureus* L.) in the eastern

Croatia. Agriculturae Conspectus Scientificus 78:245-248.

Ćirović, D., A. Penezić, M. Milenković, and M. Paunović. 2014. Winter diet composition of

the golden jackal (*Canis aureus* L., 1758) in Serbia. Mammalian Biology 79:132-127.

Chourasia, P., K. Mondal, K. Sankar, and Q. Qureshi. 2012. Food habits of golden jackals

(*Canis aureus*) and striped hyena (*Hyaena hyaena*) in Sariska Tiger Reserve, western India. World Journal of Zoology 7:106-112.

Giannatos, G., A. Karypidou, A. Legakia, and R. Polymeni. 2010. Golden jackal (*Canis aureus* L.) diet in southern Greece. Mammalian Biology 75:227-232.

Gupta, S. 2011. Ecology of medium and small sized carnivores in Sariska Tiger Reserve,

Rajasthan, India. Ph.D. dissertation, Saurashtra University, Rajkot, India.

Heptner, V. G., and N. P. Naumov. 1998. Mammals of the Soviet Union, volume II, part 1a.

Amerind Publishing Co., New Delhi, India.

Hoffmann, M., J. Arnold, J. W. Duckworth, Y. Jhala, J. F. Kamler, and M. Krofel. 2018. *Canis*

*aureus*. The IUCN Red List of Threatened Species 2018:e.T118264161A46194820. [www.iucnredlist.org](http://www.iucnredlist.org). Accessed on 10 July 2020.

Jeager, M. M., E. Haque, P. Sultana, and R. L. Bruggers. 2007. Daytime cover, diet and space-use of golden jackals (*Canis aureus*) in agro-ecosystems of Bangladesh. Mammalia 71:1-10.

Khan, A. A., and M. A. Beg. 1986. Food of some mammalian predators in the cultivated areas of Punjab. Pakistan Journal of Zoology 18:71-79.

Khan, K. A., J. A. Khan, and N. Mohan. 2017. Winder food habits of the golden jackal *Canis*

*aureus* (Mammalia: Carnivora: Canidae) in Patna Bird Sanctuary, Uttar Pradesh, India. Journal of Threatened Taxa 9:10656-10661.

Lanszki, J., G. Giannatos, A. Dolev, G. Bino, and M. Heltai. 2010. Late autumn trophic

flexibility of the golden jackal *Canis aureus*. Acta Theriologica 55:361-370.

Lanszki, J., G. Giannatos, M. Heltai, and A. Legakis. 2009. Diet composition of golden

jackals during cub-rearing season in Mediterranean marshland in Greece. Mammalian Biology 74:72-75.

Lanszki, J., M. W. Hayward, and N. Nagyapáti. 2018. Feeding responses of the golden jackal

after reduction of anthropogenic food subsidies. PLoS ONE 13:e0208727.

Lanszki, J., and M. Heltai. 2002. Feeding habits of golden jackal and red fox in south-western

Hungary during winter and spring. Mammalian Biology 67:129-136.

Lanszki, J., M. Heltai, and L. Szabó. 2006. Feeding habits and trophic niche overlap between

sympatric golden jackal (*Canis aureus*) and red fox (*Vulpes vulpes*) in the Pannonian ecoregion (Hungary). Canadian Journal of Zoology 84:1647-1656.

Lanszki, J., A. Kurys, M. Heltai, S. Csányi, and K. Ács. 2015. Diet composition of the golden

jackal in an area of intensive big game management. Annales Zoologici Fennici 52:243-255.

Lanszki, J., A. Kurys, L. Szabó, N. Nagyapáti, L. B. Porter, and M. Heltai. 2016. Diet

composition of the golden jackal and sympatric red fox in an agricultural area (Hungary). Folia Zoologica 65:310-322.

Macdonald, D. W. 1979. The flexible social system of the golden jackal, *Canis aureus*.

Behavioral Ecology and Sociobiology 5:17-38.

Mahmood, T., F. Niazi, and M. S. Nadeem. 2013. Diet composition of Asiatic jackal (*Canis*

*aureus*) in Margallah Hills National Park, Islamabad, Pakistan. Journal of Animal and Plant Sciences 23:444-456.

Majumder, A., K. Sankar, Q. Qureshi, and S. Basu. 2011. Food habits and temporal activity

patterns of the golden jackal *Canis aureus* and the jungle cat *Felis chaus* in Pench Tiger Reserve, Madhya Pradesh, India. Journal of Threatened Taxa 3:2221-2225.

Markov, G., and J. Lanszki. 2012. Diet composition of the golden jackal, *Canis aureus*, in an

agricultural environment. Folia Zoologica 61:44-48.

Mukherjee, S., S. P. Goyal, A. J. T. Johnsing, and M. R. P. Leite Pitman. 2004. The

importance of rodents in the diet of jungle cat (*Felis chaus*), caracal (*Caracal caracal*) and golden jackal (*Canis aureus*) in Sariska Tiger Reserve, Rajasthan, India. Journal of Zoology 262:405-411.

Nadeem, M., et al. 2012. Season- and locality-related changes in the diet of Asiatic jackal

(*Canis aureus*) in Potohar, Pakistan. Turkish Journal of Zoology 36:798-805.

Poché, R. M., S. J. Evans, P. Sultana, M. E. Hague, R. Sterner, and M. A. Siddique. 1987.

Notes on the golden jackal (*Canis aureus*) in Bangladesh. Mammalia 51:259-270.

Prerna, S., A. Edgaonkar, and Y. Dubey. 2015. Status of golden jackal *Canis aureus* and

ungulates in a small enclosed area – Van Vihar National Park, Madhya Pradesh, India. Journal of Threatened Taxa 7:7416-7421.

Radović, A., and D. Kovačić. 2010. Diet composition of the golden jackal (*Canis aureus* L.)

on the Pelješac Peninsula, Dalmatia, Croatia. Periodicum Biologorum 112:219-224.

Raichev, E. G., H. Tsunoda, C. Newman, R. Masuda, D. M. Georgiev, and Y. Kaneko. 2013.

The reliance of the golden jackal (*Canis aureus*) on anthropogenic foods in winter in central Bulgaria. Mammal Study 38:19-27.

Ramakumaran, K., R. Chandran, C. Satyanarayana, K. Chandra, and T. Shyamal. 2017.

Density and obligatory feeding habits of an isolated golden jackal *Canis aureus* L. (Mammalia: Carnivora: Canidae) population in Pirotan Island, Gulf of Kachchh, India. Journal of Threatened Taxa 9:10121-10124.

Rice, C. G. 1986. Observations on predators and prey at Eravikulam National Park, Kerala.

Journal of the Bombay Natural History Society 83:283-305.

Sankar, K. 1988. Some observations on food habits of jackals (*Canis aureus*) in Keoladeo

National Park, Bhatapur, as shown by scat analysis. Journal of the Bombay Natural History Society 85:185-186.

Schaller, G. B. 1967. The deer and the tiger: a study of wildlife in India. The University of

Chicago Press, Chicago, Illinois, USA.

Schaller, G. B. 1977. Mountain monarchs: wild sheep and goats of the Himalaya. The

University of Chicago Press, Chicago, Illinois, USA.

Shabbir, S., M. Anwar, I. Hussain, and M. A. Nawaz. 2013. Food habits and diet overlap of

two sympatric carnivore species in Chitral, Pakistan. Journal of Animal and Plant Sciences 23:100-107

Singh, A., A. Mukherjee, S. Dookia, and H. N. Kumara. 2016. High resource availability and lack of competition have increased population of a meso-carnivore – a case study of the golden jackal in Keoladeo National Park, India. Mammal Research 61:209-219.

Tsunoda, H., S. Peeva, E. Raicheve, K. Ito, and Y. Kaneko. 2019. Autumn dietary overlaps among three sympatric mesocarnivores in the central park of Stara Planina Mountain, Bulgaria. Mammal Study 44:275-281.

Tsunoda, H., E. G. Raicheve, C. Newman, R. Masuda, D. M. Georgiev, and Y. Kaneko. 2017. Food niche segregation between sympatric golden jackals and red foxes in Bulgaria. Journal of Zoology 303:64-71.

Volozheninov, N. N. 1972. Feeding of *Canis aureus aureus*, *Vulpes vulpes flavescens* and *Felis chaus oxianus* in South Uzbekistan. Zoologicheskii Zhurnal 51:1048-1053. [In Russian with English summary]
